# Second‐generation capsule endoscopy for the detection of colorectal polyps: An updated systematic review and comparative meta‐analysis of prospective studies

**DOI:** 10.1111/codi.70362

**Published:** 2026-01-20

**Authors:** Cauã Ferreira Câmara, Pedro Robson Costa Passos, Ettore Carvalho Lopes Cezar, José Nilo de Lima Filho, Rafael Mariano Araújo Oliveira, Carlos Yuri Monteiro de Paiva, Adriely Oliveira Quintela, Alana Ferreira de Andrade, Lara Burlamaqui Veras

**Affiliations:** ^1^ Universidade Federal do Ceará Fortaleza Brazil; ^2^ Hospital Universitário Walter Cantídio Fortaleza Brazil

**Keywords:** Bayesian bivariate model, colon capsule endoscopy, colorectal cancer screening, colorectal polyps, decision curve analysis, diagnostic accuracy, meta‐analysis

## Abstract

**Objectives:**

Colorectal cancer (CRC) is the third most common cancer worldwide and the second leading cause of cancer‐related death. Colonoscopy (CO) is the gold standard for screening, but its invasive nature and low adherence limit its use. Second‐generation capsule endoscopy (CCE‐2) emerges as a non‐invasive alternative. This study aimed to evaluate the diagnostic accuracy of CCE‐2 in detecting colorectal polyps, using CO as the reference standard.

**Methods:**

A systematic review and meta‐analysis of prospective studies in adults undergoing CCE‐2 followed by CO was performed. Searches were conducted in PubMed, EMBASE and Cochrane up to May 2025. Diagnostic accuracy metrics were pooled using a Bayesian bivariate model with construction of HSROC curves. To assess clinical impact, a fictitious cohort of 1000 patients was simulated based on polyp prevalence and submitted to decision curve analysis (DCA).

**Results:**

Twelve studies including 4316 patients were analysed. CCE‐2 demonstrated sensitivity/specificity of 0.89/0.94 for polyps ≥6 mm and 0.91/0.98 for ≥10 mm. In FIT‐positive patients, performance was superior. In the simulated cohort, CCE‐2 showed greater net benefit from a 20% pre‐test probability threshold of a patient to have a polyp of any size, surpassing the universal CO strategy, avoiding unnecessary colonoscopies to make this diagnostic.

**Conclusion:**

CCE‐2 is a non‐invasive, safe and accurate alternative for CRC screening, with potential to increase adherence, particularly in patients with contraindications or refusal of CO. However, the lack of therapeutic capability and absence of evidence regarding mortality reduction limit its role as a first‐line tool. Randomized clinical trials are needed to consolidate its role in personalized screening.

**What does this paper add to the literature?**

This study provides the most comprehensive and methodologically robust evaluation of CCE‐2 diagnostic accuracy to date, incorporating Bayesian bivariate modelling and decision curve analysis. It demonstrates its net clinical benefit in personalized screening strategies and highlights superior performance in FIT‐positive individuals.

## INTRODUCTION

Colorectal cancer (CRC) is the third most common cancer worldwide and the second leading cause of cancer‐related death, with a significant burden in both incidence and mortality [1, 2]. In its early stages, CRC typically progresses without noticeable symptoms, making early detection challenging in the absence of organized screening programmes [3]. Screening plays a vital role in identifying malignant lesions and adenomatous polyps, recognized precursors to CRC [4], at an earlier, more treatable stage. Timely detection significantly improves treatment outcomes, often enabling less invasive and more cost‐effective interventions [5, 6].

The methods used for colorectal cancer screening—such as the faecal occult blood test (FOBT), faecal DNA test, sigmoidoscopy and full or virtual colonoscopy—may vary according to geographic region, reflecting differences in public policies, resource availability and population adherence. A recent review highlighted these variations across countries, emphasizing that the choice of screening method is often linked to economic, cultural and structural factors within the healthcare system [7].

Colonoscopy (CO) is considered the gold standard method for CRC screening [8]; however, its invasive nature and the potential risks associated with the procedure limit its use in certain patient groups. CO is associated with a higher risk of complications compared to noninvasive screening modalities, including perforation (approximately 0.5 per 1000), postprocedural bleeding (2.6 per 1000, primarily after polypectomy), aspiration pneumonitis (especially with deep sedation) and death (2.9 per 100,000) [9]. Additionally, the need for sedation can be a barrier, especially in individuals with comorbidities, in which the risk–benefit ratio becomes less favourable [10, 11].

A widely used diagnostic method is the faecal immunochemical test (FIT), which detects the presence of occult blood in spontaneous stool samples through an antibody specific to the globin portion of haemoglobin. Although FIT demonstrates good sensitivity and specificity, its performance can be affected by the stage of colorectal cancer as well as the location and size of adenomas. It also shows poor or no sensitivity for serrated precursor lesions and often requires repeated testing, which can be problematic in non‐programmatic settings [8]. However, it is common for individuals with a positive FIT result to undergo diagnostic colonoscopy and subsequently not present with adenomas or significant disease, leading to unnecessary colonoscopies. This scenario increases the burden and cost of healthcare services, exposes patients to avoidable procedures, may reduce adherence to annual FIT‐based screening and can cause psychological distress [3].

In this context, capsule endoscopy emerges as a non‐invasive alternative with the potential to effectively detect colorectal lesions with a lower risk of complications [12, 13]. In recent years, second‐generation capsule endoscopy (CCE‐2) has been investigated in prospective studies as a screening tool for the detection of colorectal polyps [14, 15]. With significant improvements in image quality, procedure time, and patient adherence rates, CCE‐2 may offer a safer and more acceptable solution, especially for those with contraindications to CO [15]. Furthermore, ongoing research is focused on integrating artificial intelligence for automated lesion detection and further expanding the diagnostic reach of capsule endoscopy [16].

Despite these advances, the diagnostic accuracy of CCE‐2 in detecting polyps of varying sizes still requires rigorous comparative evaluation against CO. Previous systematic reviews on this topic presented important limitations, such as small sample sizes, restricted non‐Bayesian statistical analyses, lack of heterogeneity assessment and absence of pre‐registered protocols, all of which compromise methodological robustness and the reproducibility of results [17, 18].

Therefore, the objective of this article is to present an updated systematic review and comparative meta‐analysis of the best available evidence assessing the diagnostic accuracy of CCE‐2 in detecting colorectal polyps, using CO as the reference standard.

## MATERIALS AND METHODS

### Protocol registration

This study was conducted and reported according to the 2020 Preferred Reporting Items for Systematic Reviews and Meta‐Analyses (PRISMA) guidelines (Table S1) [19]. The protocol was registered in the International Prospective Register of Systematic Reviews database under protocol CRD420251035437.

### Search strategy and study selection

We systematically searched PubMed, the Cochrane Central Register of Controlled Trials and EMBASE for human cohort studies published up to May 2025. The complete search strategy is provided in Table S2. Studies were eligible for inclusion if they met the following criteria: (i) involved adult participants (≥18 years; to avoid paediatric studies) undergoing CCE‐2 for CRC screening; (ii) included prospective follow‐up conventional CO performed on the same individuals; and (iii) reported sufficient data to allow estimation of diagnostic performance metrics (e.g. sensitivity, specificity). We excluded studies with low levels of evidence (e.g. retrospective studies, case series, case reports, conference abstracts), studies involving individuals with a prior diagnosis of CRC or hereditary polyposis syndromes, studies in which CO was performed before CCE‐2 (preventing the assessment of false‐positives) and those employing experimental methods (e.g. artificial intelligence or chromatography) that precluded direct comparison with standard approaches.

### Data extraction

Two investigators independently screened titles and abstracts, followed by an analysis of full‐text articles meeting predefined criteria. Discrepancies in inclusion criteria were resolved by a third investigator. Data extracted from eligible studies included the following: (i) general characteristics (first author, publication year, study design, sample size, mean follow‐up, mean age, male percentage, results and conclusions); (ii) relevant diagnostic metrics; and (iii) number of true‐positives, false‐positives, true‐negatives and false‐negatives. For studies lacking mean or standard values, we estimated them using the median, range, interquartile range, and sample size via the Box‐Cox approach and the methods described by MacGrath et al. (2021) [20, 21].

### Assessment of quality

Study quality was independently assessed by two reviewers using the QUADAS‐2 tool, which is designed for the evaluation of diagnostic accuracy studies. This tool examines four domains: patient selection, index test, reference standard and flow and timing. Each domain is evaluated for risk of bias, and the first three domains are also assessed for concerns regarding applicability [22]. An overall risk of bias was classified as ‘high’ if any domain was rated as high risk, and as ‘unclear’ if no domains were high risk but at least one was rated as unclear. Any discrepancies between reviewers were resolved through consultation with a third investigator.

### Outcomes

The main outcomes of this diagnostic accuracy meta‐analysis were the sensitivity and the specificity of CCE‐2 for the detection of colorectal polyps, using CO as the standard. True‐positives and true‐negatives were defined as the presence or absence of polyps firstly identified by CCE‐2 and then confirmed by CO, while false‐positives and false‐negatives were defined as a discordance between the CCE‐2 initial results and the following CO definitive assessment. Secondary outcomes included the incomplete examination rate of CCE‐2 (e.g. capsule not reaching the rectum or incomplete visualization of the colon), expressed as a proportion, bowel preparation adequacy, as defined by the original studies (e.g. excellent/good vs. fair/poor) and its impact on diagnostic performance and adverse events related to CCE‐2.

### Data synthesis

The number of true‐positive, false‐positive, true‐negative and false‐negative detections by CCE‐2 were directly extracted from each study (or calculated using specificity and sensitivity) and turned into sensitivity and specificity using standard methods [23]. For pooling, we employed a Bayesian bivariate framework that jointly models sensitivity and specificity, accounting for their correlation and between‐study heterogeneity. This model is also well‐suited to handle data missingness [24]. Weakly informative priors were used to stabilize estimation while maintaining flexibility. Model convergence was assessed through inspection of R‐hat statistics, effective sample sizes and trace plots, ensuring the reliability of the posterior estimates. Model fit was evaluated using posterior predictive checks, and sensitivity analyses were conducted to examine the impact of prior specifications and influential studies. All analyses were conducted in R software (Version 4.3.2, R Foundation for Statistical Computing, Vienna, Austria) using the ‘brms’ and ‘dca’ packages.

### Additional analyses

Linear or restricted cubic spline regression (with three knots defined by percentiles) and subgroup analyses to elucidate potential effect moderators [25]. As Bayesian hierarchical models ‘shrink’ the study‐level estimates towards the group mean (reducing between‐study variance aggressively) [26], regression analyses were conducted based on frequentist estimates (as previously calculated or directly extracted from studies). To improve interpretability and clinical applicability, we constructed hierarchical summary receiver operating characteristic (HSROC) curves and conducted decision curve analysis (DCA) to characterize the net benefit of CCE‐2 across a range of threshold probabilities [27]. In this context, net benefit represents the balance between the true‐positive findings gained by using CCE‐2 and the false‐positive findings leading to unnecessary colonoscopies, after weighting these outcomes by the threshold probability that reflects the clinician's or patient's acceptable trade‐off between missing a lesion and performing an avoidable invasive test. The robustness of findings was further supported by assessing potential small‐study effects and publication bias through visual inspection of funnel plots of the log‐transformed frequentist diagnostic odds ratio (DOR) and formal statistical testing through Deek's funnel plot asymmetry test.

## RESULTS

### Search results

**FIGURE 1:**
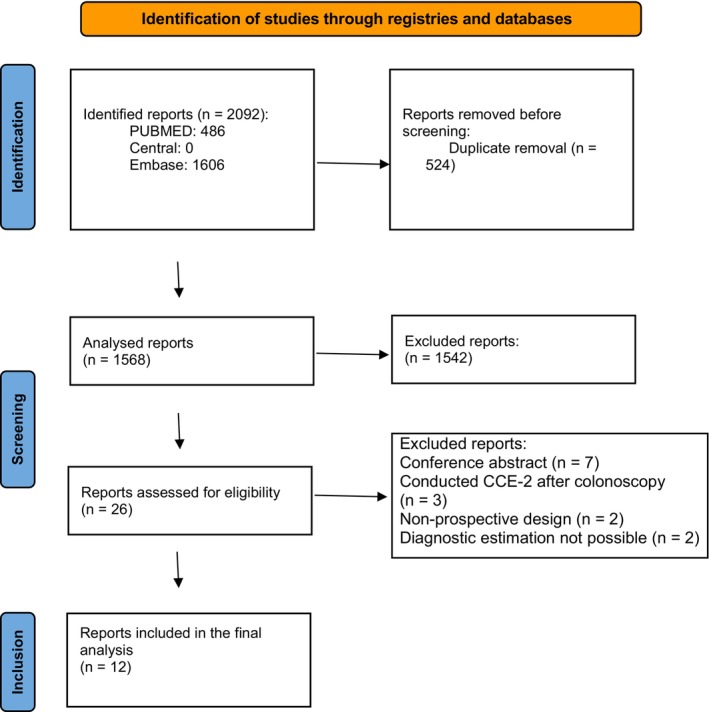
PRISMA flow chart exhibiting the steps taken in study selection.

Out of the initial 2092 articles yielded by the search string, 1568 remained after excluding duplicates. After title and abstract screening, 26 reports were chosen to be evaluated in full text. Of these, seven were conference abstracts, three conducted CCE‐2 after CO, two were not prospective and two did not make enough data for diagnostic estimation available, precluding their inclusion. Finally, 12 full articles [28, 29, 30, 31, 32, 33, 34, 35, 36, 37, 38, 39] were included in our analysis. The PRISMA flow diagram can be seen in Figure 1.

### Main characteristics of the included studies

Of the 12 included studies, all were observational prospective studies evaluating CCE‐2 with follow‐up CO. Nine studies were specifically restricted to CRC screening conditions, of which five analysed only patients with positive faecal immunochemical testing (FIT). A total of 4316 patients across 10 countries and three continents were included. Of the 907 patients with sex data available, 507 (55.9%) were male, while the 879 patients with age data had a mean age of 59.09 years. Adequate bowel preparation varied from 61% to 96% of patients, with most bowel preparation protocols including polyethylene glycol (PEG 2L, 11/13) and clear liquid diets (11/13). Furthermore, six studies used senna tablets, four utilized sodium phosphate and three used bisacodyl in their protocols. All but two studies included patients with incomplete transit in diagnostic yield analysis. Eight studies were classified as having low risk of bias, while two had unclear assessments and two were classified as having high risk of bias (see Figure S1). See Table 1 for full details on the included studies.

**TABLE 1:** Main characteristics of the included studies.

Year	Author	Study design	Country	Sample size	Clinical context	% Male/no. male	Mean age (SD)	Patients with polyps detected (≥6 mm/≥10 mm)	Polyps detected (≥6 mm/≥10 mm) (reference method)	Bowel preparation protocol	Capsule transit time (mean ± SD or median)	Complete transit (%)	Adequate preparation (%)
2014	Rodonotti	Prospective	Italy	50	Positive FIT	58% (29)	59.2 ± 5.2 years	32 total (16 ≥ 6 mm; 13 ≥ 10 mm)	82	Low‐fibre diet (2 days) + clear liquids +2 × 1 L macrogol 3350 with ascorbic acid + bisacodyl 20 mg + metoclopramide IV	213.8 ± 173.1 min (colon)	100%	70%
2021	Cash	Prospective	UK	145	Screening population	37% (53)	55.3 ± 5.4 years	90 total (44 ≥ 6 mm; 19 ≥ 10 mm)	Not reported	Clear liquids + senna tablets +2 L PEG evening before and morning	Not reported	97%	84.1%
2014	Hagel^c^	Prospective	Germany	24	Suspected CRC or known colonic disease	58% (14)	51.1 ± 13.10	Not reported^b^	44 (16 < 6 mm, 17 between 6 and 9 mm, 11 > 10 mm)	Senna tablets + liquid diet +4 × 2 L PEG + NaP boosters + bisacodyl suppository	7 h05 ± 3 h49	71%	95.8%
2018	Kobaek‐Larsen^c^	Prospective comparative	Denmark	253	Positive FIT	58% (147)	Not reported	223 (>9 mm)	434	Magnesium oxide + clear fluids +2 L Moviprep + boosters + bisacodyl suppository + domperidone	Not reported	50%	54% (only accounting the ones that excreted the capsule)^d^
2019	Voska	Prospective	Czech Republic	225	<60 years, no personal/family CRC history	Not reported	59 ± 5.59 years	114 total (34 ≥ 6 mm; 16 ≥ 10 mm)	Not reported	Low‐residue diet + clear liquids +4 L PEG (split) + NaP boosters + glycerine suppository	3 h48	89%	90.2%
2015	Rex	Prospective	USA + Israel	695	Screening population	Not reported	Not reported	192 ≥ 6 mm; 79 ≥ 10 mm	368 lesions ≥6 mm and 114 lesions ≥10 mm	Senna tablets + clear liquids +2 × 2 L PEG	Median 1h16m before, 1h39m after exclusions	100%^a^	80%
2014	Holleran	Prospective	Ireland	62	Positive FIT	55% (34)	62.5 ± 5.8 years	36 total	Not reported	Senna tablets + PEG 2 L	7 hours	74%	91.9%
2020	Pecere	Prospective	Italy	178	Positive FIT	70% (125)	61 years (no SD)	123 (98 ≥ 6 mm; 60 ≥ 10 mm)	Not reported	Senna + PEG 2 L (split dose)	4 h04	100%^a^	88.2%
2020	González‐Suárez	Prospective	Spain	145	Positive FIT	57% (83)	60.1 ± 5.8 years	112 (77 ≥ 6 mm; 37 ≥ 10 mm)	Not reported	Senna tablets + PEG 1 L evening and morning + metoclopramide	Not reported	Not reported	81.4%
2025	Turvill^c^	Prospective	UK	2301	Suspected CRC pathway, FIT ≤100 μg Hb/g faeces	Not reported	Not reported	492 (6–9 mm); 259 ≥ 10 mm	944 between 6 and 9 mm and 578 greater than 10 mm	3‐day low residue diet + split PEG + boosters (gastrografin/phosphosoda) + prucalopride	Not reported	Not reported	‐
2025	Hotta^c^	Prospective	Japan	188	Established or suspected colorectal diseases	Not reported	Not reported	125 total (86 ≥ 6 mm; 55 ≥ 10 mm)	317 (152 < 6 mm, 76 between 6 and 9 mm, 78 > 10 mm)	Low fibre diet + magnesium citrate + laxatives + MoviPrep + mosapride	Not reported	Not reported	76%
2016	Morgan	Prospective	USA	50	Patients 18–70 with indication for colonoscopy	44% (22)	60.2 ± 8.47 years	22 total (15 ≥ 6 mm; 7 ≥ 10 mm)	116 (66 ≤ 5 mm, 22 between 6 and 9 mm, 11 > 10 mm)	4 L PEG split dose (2 L evening before +2 L morning)	Not reported	64%	61%

Abbreviations: CRC, colorectal cancer; FIT, faecal immunochemistry test; PEG, polyethylene glycol; SD, standard deviation.

^a^ In their analysis, these studies considered incomplete transit as an exclusion criterion, leading to all included patients having complete transit.

^b^ As this study did not report the number of patients with polyps, it was not included in per‐patient analysis.

^c^ As only polyps >9 mm were included, this study was not included in the overall diagnostic accuracy analysis, but was included in the >10 mm polyp analysis.

^d^ As we included the estimates that encompassed patients with incomplete transit in our main analysis, this percentage was not used in meta‐regression.

### Per‐patient diagnostic performance

**FIGURE 2:**
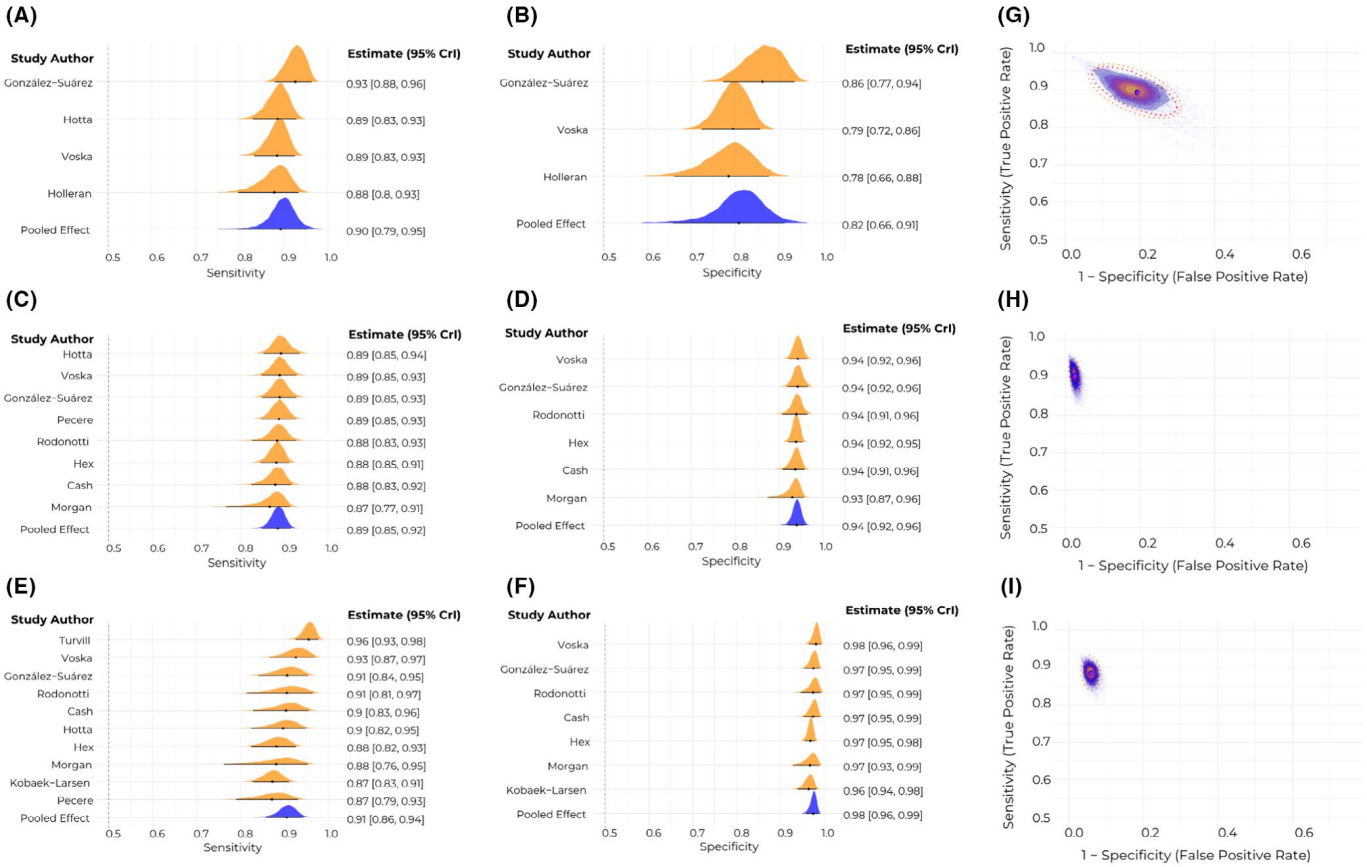
Diagnostic metrics in the per‐patient analysis. Pooled estimates for sensitivity and specificity of polyps of all sizes (A and B, respectively), polyps with 6 mm or larger (C and D, respectively) and polyps with 10 mm or larger (E and F, respectively) were pooled based on a Bayesian bivariate model. Hierarchical summary receiver operating characteristic curves for polyps of all sizes, those with 6 mm or larger and those with 10 mm or larger can be seen in (G), (H) and (I), respectively. CrI, credible interval.

Among the only four studies reporting per‐patient diagnostic performance for polyps of any size, the estimated sensitivity was 0.90 (95% CrI: 0.79–0.95, Figure 2A) and the specificity was 0.82 (95% CrI: 0.66–0.91, Figure 2B). For polyps measuring 6 mm or larger, based on data from 8 studies, the estimated sensitivity was 0.89 (95% CrI: 0.85–0.92, Figure 2C) and the specificity was 0.94 (95% CrI: 0.92–0.96, Figure 2D). Among the 10 studies evaluating polyps 10 mm or larger, the estimated sensitivity reached 0.91 (95% CrI: 0.86–0.94, Figure 2E) and the specificity was 0.98 (95% CrI: 0.96–0.99, Figure 2F). The results of subgroup analysis can be seen in Table 2. Of note, FIT positivity seemed to increase sensitivity and decrease specificity. The results of regression analyses based on moderators are displayed on Table S3, of which the only significant moderator was the proportion of adequate cleansing on the sensibility to detect polyps 10 mm or larger on restricted cubic spline (*p* = 0.040). Upon further testing, this relationship was found to be linear (*p* = 0.047, *R* ^2^ = 0.510, Figure S2). HSROC curves can be seen in Figure 2G–I.

**TABLE 2:** As subgroup analyses generally have lower statistical power than standard analyses, we increased the number of iterations and employed a slightly more informative prior to improve model convergence and estimation stability.

Subgroup		All sizes			≥ 6 mm			≥ 10 mm		
		Sensitivity (95% CrI)	Specificity (95% CrI)	Posterior probability of the interaction effect being >0	Sensitivity (95% CrI)	Specificity (95% CrI)	Posterior probability of the interaction effect being >0	Sensitivity (95% CrI)	Specificity (95% CrI)	Posterior probability of the interaction effect being >0
Faecal immunochemistry test results	Positive	0.97 (0.40–1.00)	0.71 (0.01–1.00)	0%	0.93 (0.79–0.98)	0.87 (0.44–0.99)	0.00625%	0.93 (0.67 0.99)	0.95 (0.51–1.00)	0.3125%
	Not explicit	0.85 (0.52–0.97)	0.88 (0.58–0.97)		0.87 (0.81–0.92)	0.95 (0.93–0.97)		0.88 (0.77–0.95)	0.98 (0.96–0.99)	
Adenomas only^a^		–	–	–	0.87 (0.40–0.97)	–	–	0.91 (0.54–0.99)	–	–
Continent	Europe	0.91 (0.44–0.99)	0.83 (0.00–1.00)	–	0.89 (0.68–0.96)	0.95 (0.71–0.99)	81.43125%	0.92 (0.62–0.99)	0.98 (0.67–1.00)	33.525%
	Not Europe^b^	–	–		0.88 (0.81–0.93)	0.93 (0.89–0.96)		0.87 (0.74–0.95)	0.97 (0.93–0.99)	
Transit^c^	Complete in all patients^d^	–	–	–	0.87 (0.49–0.98)	0.94 (0.48–1.00)	13.225%	0.92 (0.54–0.99)	0.98 (0.57–1.00)	41.08125%
	Not complete in all patients	–	–		0.82 (0.67–0.91)	0.95 (0.89–0.97)		0.89 (0.77–0.96)	0.98 (0.94–1.00)	

^a^ As no studies reported the specificity specifically for adenomas, the estimations were based on a non‐bivariate Bayesian model.

^b^ As only non‐European study reported both diagnostic metrics for polyps of all sizes, subgroup analysis on this category was not feasible.

^c^ As none of the studies with 100% transit rates included assessment for polyps of all sizes, this analysis was not conducted.

^d^ This analysis included a subgroup analysis from Kobaek‐Larsen et al. (2017) that had not been included in our main analysis.

### Per‐polyp diagnostic performance

**FIGURE 3:**
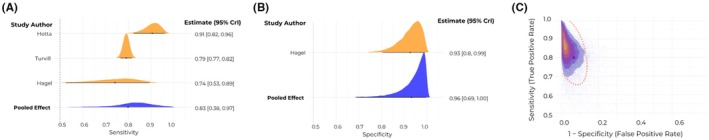
Diagnostic metrics in the per‐polyp analysis. Pooled estimates for sensitivity and specificity of polyps between 6 and 9 mm can be seen in (A) and (B), respectively, and were the only estimates pooled for this analysis. Hierarchical summary receiver operating characteristic curve for polyps between 6 and 9 mm can be seen in (C). CrI, credible interval.

Considering the scarcity of per‐polyp data, the only analysis that could be reliably estimated within the Bayesian model without divergences was the analysis of polyps between 6 and 9 mm of size, encompassing three studies. In this analysis, the sensitivity was 0.83 (95% CrI 0.38–0.97), while the specificity reached 0.96 (95% CrI 0.69–1.00). The limited number of studies precluded effective subgroup or regression modelling. The HSROC curve can be seen in Figure 3C.

### Publication bias

Publication bias was assessed only on per‐patient DOR, as it composed our main analysis. Upon visual examination of the funnel plots, no clear sign of publication bias was noted (Figure S3). Furthermore, Denk's publication bias test yielded no evidence of bias (*p* = 0.49, 0.96 and 0.19 for polyps of any size, ≥6 and ≥10 mm, respectively).

### Decision curve analysis

**FIGURE 4:**
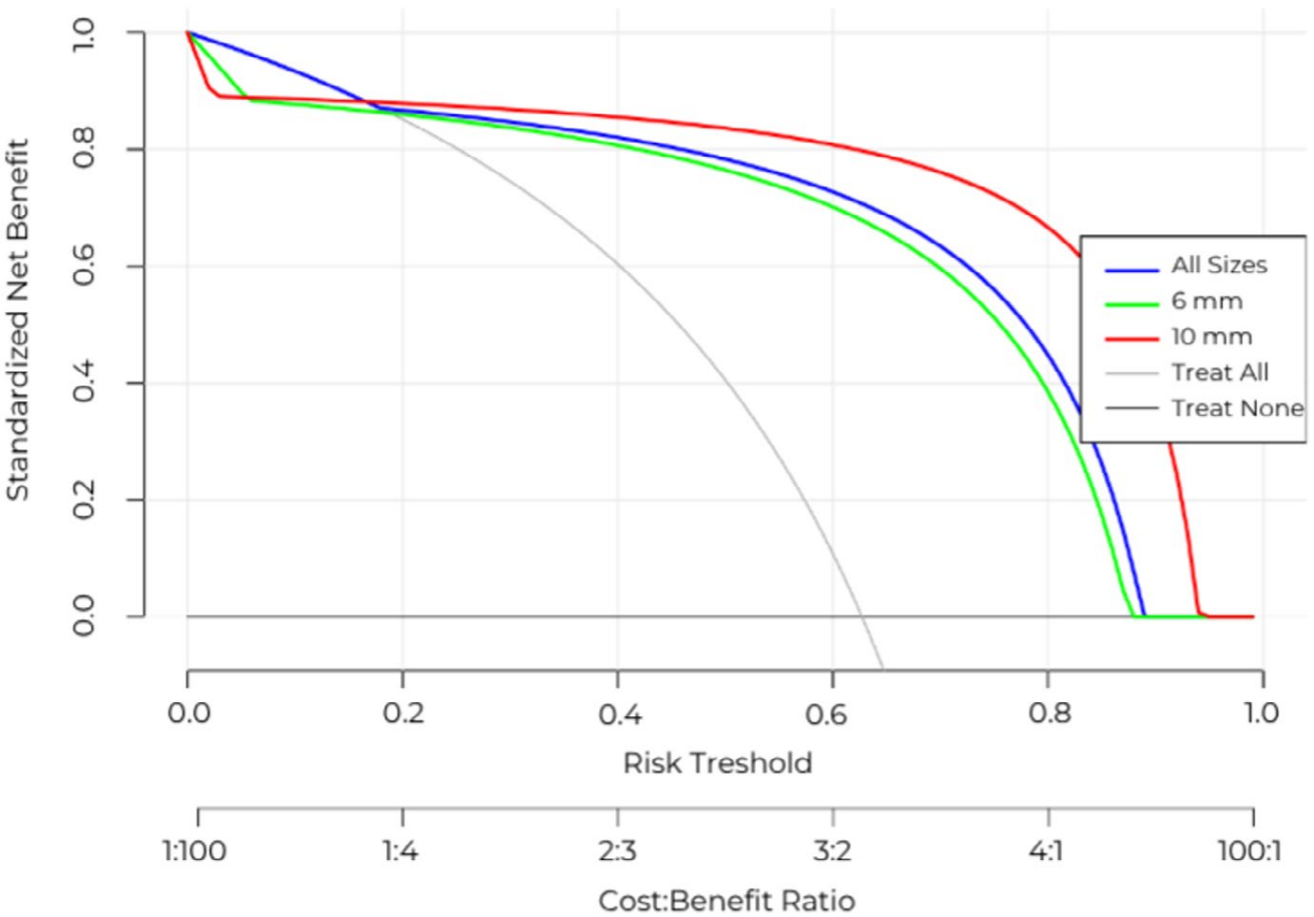
Decision curve analysis based on simulated data from a hypothetical cohort of 1000 patients undergoing polyp screening. Diagnostic performance metrics of CCE‐2 for each polyp size category were derived from posterior predictions obtained through meta‐analytic pooling, simulating results for a new, unseen study. Polyp prevalences by size were aggregated from the included studies and incorporated into this simulation. The *x*‐axis ‘cost–benefit ratio’ refers to the decision‐analytic trade‐off between the harm of an unnecessary colonoscopy and the benefit of correctly detecting a polyp and should not be interpreted as a monetary ratio.

To evaluate the performance of CCE‐2 and its net benefit against colonoscopy, considering the number of patients who would undergo an unnecessary colonoscopy, we simulated an artificial screening cohort of 1000 patients. This simulation incorporated the nested prevalence of polyps by size, calculated based on data from all included studies, using diagnostic metric values predicted for a new study through Bayesian per‐patient modelling (see Table S4). To interpret these results, we conducted a DCA to assess the net benefit depending on the probability of a patient to have a polyp of any size (Figure 4). Notably, up to a 17.7% risk threshold for polyps of any size, CCE‐2 showed a net benefit equivalent to the strategy of treating (in this context, undergoing CO) all patients. Beyond this threshold, however, CCE‐2 demonstrated superior net benefit across all higher probabilities. Specifically, starting at a 20% threshold probability, CCE‐2 provided a greater net benefit for screening polyps of all sizes, reducing the number of unnecessary invasive procedures, as well as those >6 and > 10 mm, with this benefit persisting until the threshold surpassed approximately 90%, where it eventually diminished to zero.

## DISCUSSION

In this meta‐analysis of prospective data encompassing over 4000 patients, CCE‐2 demonstrated excellent diagnostic performance for detecting colorectal polyps, with accuracy increasing proportionally with polyp size. Moreover, our DCA highlights that while universal CO referral may be justifiable in patients with very low pre‐test probabilities of polyps, CCE‐2 provides greater net clinical benefit in those at lower risk, supporting its role in personalized, risk‐adapted screening strategies.

Given the rising incidence of CRC and the poor prognosis associated with late‐stage diagnosis, screening is universally recommended beginning at age 45, with earlier initiation in high‐risk groups [40, 41]. Nonetheless, CO is an invasive procedure with relative contraindications including severe or advanced cardiopulmonary disease and advanced age [4, 42, 43]. Furthermore, adherence to CO remains suboptimal due to invasiveness, fear of discomfort and logistical constraints. In this context, several studies indicate that patients strongly prefer capsule‐based screening modalities over endoscopic alternatives, with a prospective study displaying significantly improved adherence (up to fourfold) when CCE is offered as an option [44, 45, 46].

Cost remains a central challenge to broader CCE‐2 adoption, although their potential benefit on patient compliance may surpass this disadvantage. While CO is generally more cost‐effective when adherence is equal, modelling studies suggest that CCE‐2 becomes economically favourable when it improves adherence by 20–30% [47]. A major limitation is the lack of evidence showing that CCE‐2‐based screening reduces CRC mortality, and unlike CO, it does not allow for immediate therapeutic intervention. Thus, while further studies on CCE‐2 based screening should be conducted to investigate potential advantages, current guidelines from major U.S. bodies, including the US Multi‐Society Task Force and the American Gastroenterological Association, do not recommend CCE‐2 for first‐line screening [9, 41]. Nonetheless, CCE‐2 is approved in Europe as a screening option for average‐risk individuals and high‐risk patients unable or unwilling to undergo CO [48].

Despite its promising capabilities, CCE‐2 is not currently endorsed for CRC screening by major regulatory bodies, including the U.S. Food and Drug Administration (FDA) [49]. Consequently, patients who are unwilling or unable to undergo CO are often limited to non‐invasive options such as stool‐ or blood‐based tests [50]. However, many of these alternatives, such as the FIT and multitarget stool DNA testing, exhibit suboptimal sensitivity, particularly in detecting early‐stage lesions [51, 52, 53]. Additionally, these tests do not offer etiological insights, and a positive result still necessitates follow‐up CO [8]. In our subanalysis of FIT+ patients, CCE‐2 demonstrated excellent sensitivity in identifying both early‐ and late‐stage polyps (all at 93% or higher), with adequate specificity. This suggests that sequential testing with CCE‐2 in FIT+ patients may be as efficient as CO follow‐up and with potentially better adherence, as compliance of FIT+ subjects to post‐FIT CO remains suboptimal and varies from less than 50% to 90% within 1 year of a positive test [35, 54]. Of note, CE has shown superior diagnostic accuracy to computed tomography colonography in patients unwilling or with incomplete CO [55, 56]. Moreover, in FIT‐negative individuals, CCE‐2 also outperformed known FIT sensitivity estimates [8, 57, 58] and displayed excellent specificity, indicating that patients at moderate to high risk for CRC may benefit from bypassing FIT and proceeding directly to CCE‐2. Indeed, our DCA analysis displayed high net benefits for CCE‐2 in patients with higher risks of colorectal lesions. However, this approach warrants further investigation.

This meta‐analysis has limitations that must be considered. First, although all included studies were prospective and of good quality, none were RCTs, and as such, potential biases may hinder our analysis. Second, not all studies reported important variables (e.g. mean age, number of males) necessary to conduct a robust heterogeneity assessment. Finally, the context of the studies varied significantly, which hinders our capability to give general recommendations and pointed insights.

In conclusion, we have shown that CCE‐2 represents a promising adjunct in the CRC screening landscape, offering a non‐invasive alternative for patients who decline or are unsuitable for CO. While it lacks the therapeutic capacity of CO and has not yet been proven to reduce CRC mortality, its high sensitivity, particularly in FIT‐negative patients individuals, supports its potential role in sequential screening strategies. Although some studies have reported improved patient adherence and preference for capsule‐based screening compared with colonoscopy [43, 44, 45, 46], adherence itself was not directly compared in our analysis; therefore, this statement should be interpreted with caution. Regarding FIT‐negative patients, current evidence does not clearly establish CCE‐2 as a first‐line screening test. Nevertheless, additional studies should evaluate its performance in moderate‐to‐high risk populations, as CCE‐2 may offer a balance between higher sensitivity than FIT and greater acceptability than CO.

## AUTHOR CONTRIBUTIONS

**Cauã Ferreira Câmara:** Writing – review and editing; writing – original draft; conceptualization; supervision. **Pedro Robson Costa Passos:** Methodology; investigation; writing – review and editing; data curation. **Ettore Carvalho Lopes Cezar:** Writing – original draft; writing – review and editing. **José Nilo de Lima Filho:** Writing – original draft; writing – review and editing. **Rafael Mariano Araújo Oliveira:** Writing – original draft; writing – review and editing. **Carlos Yuri Monteiro de Paiva:** Writing – original draft; writing – review and editing. **Adriely Oliveira Quintela:** Writing – original draft; writing – review and editing. **Alana Ferreira de Andrade:** Writing – review and editing. **Lara Burlamaqui Veras:** Writing – review and editing; supervision; project administration.

## FUNDING INFORMATION

This research received no specific grant from any funding agency in the public, commercial or not‐for‐profit sectors.

## CONFLICT OF INTEREST STATEMENT

The authors declare no conflict of interest.

## ETHICS STATEMENT

No, there were no human subjects.

## Supporting information


Appendix S1.

## Data Availability

The data utilized in this study are accessible upon reasonable request to the corresponding author.
