# Supplementary material for: Second‐generation capsule endoscopy for the detection of colorectal polyps: An updated systematic review and comparative meta‐analysis of prospective studies
Source: Colorectal Dis. 2026 Jan 20;28(1):e70362. doi: 10.1111/codi.70362 (PMC12817651; doi:10.1111/codi.70362)
Supplement: Supplementary file 1 — Appendix S1. [file CODI-28-0-s001.docx]

**ONLINE SUPPLEMENT**

**Second-Generation Capsule Endoscopy for the Detection of Colorectal Polyps: An Updated Systematic Review and Comparative Meta-Analysis of Prospective Studies**

**Authors**: Cauã Ferreira Câmara¹; Pedro Robson Costa Passos¹; Ettore Carvalho Lopes Cezar¹; José Nilo de Lima Filho¹; Rafael Mariano Araújo Oliveira¹; Carlos Yuri Monteiro de Paiva¹; Adriely Oliveira Quintela¹; Alana Ferreira de Andrade²; Lara Burlamaqui Veras².

¹Universidade Federal do Ceará, Fortaleza – CE.

²Hospital Universitário Walter Cantídio, Fortaleza - CE.

**SUMMARY**

[**SUPPLEMENTARY TABLE S1 - PRISMA checklist. 3**](#_3znysh7)

[**SUPPLEMENTARY TABLE S2 - Search string for database search. 7**](#_x0t9u24kchjh)

[**SUPPLEMENTARY FIGURE S1 - QUADAS-2 bias assessment for included studies in traffic light (A) and summary (B) graphs. 9**](#_x8xn198u4zu6)

[**SUPPLEMENTARY TABLE S3 - Moderator analyses for per-patient diagnostic metrics based on continuous moderators 10**](#_3l7o7t996shc)

[**SUPPLEMENTARY FIGURE S2 - Plot displaying the linear relationship between the sensibility of CCE-2 for the detection of polyps with 10 mm or larger and the proportion of adequate preparation in the included studies. 11**](#_h0piuf3a1t43)

[**SUPPLEMENTARY FIGURE S3 - Funnel plots assessing publication bias based on diagnostic odds ratio for each size category. 12**](#_31t84w46w507)

[**SUPPLEMENTARY TABLE S4 - Data used for the construction of the simulated polyp screening cohort. 13**](#_2cg99v5c89as)

#

# **SUPPLEMENTARY TABLE S1 - PRISMA checklist.**

| **Section and Topic** | **Item #** | **Checklist item** | **Location where item is reported** |
| --- | --- | --- | --- |
| **TITLE** | | |  |
| Title | 1 | Identify the report as a systematic review. | Page 1 |
| **ABSTRACT** | | |  |
| Abstract | 2 | See the PRISMA 2020 for Abstracts checklist. | Page 2 |
| **INTRODUCTION** | | | ‘ |
| Rationale | 3 | Describe the rationale for the review in the context of existing knowledge. | Pages 2-3 |
| Objectives | 4 | Provide an explicit statement of the objective(s) or question(s) the review addresses. | Pages 2-3 |
| **METHODS** | | |  |
| Eligibility criteria | 5 | Specify the inclusion and exclusion criteria for the review and how studies were grouped for the syntheses. | Page 4 |
| Information sources | 6 | Specify all databases, registers, websites, organisations, reference lists and other sources searched or consulted to identify studies. Specify the date when each source was last searched or consulted. | Page 4 |
| Search strategy | 7 | Present the full search strategies for all databases, registers and websites, including any filters and limits used. | Pages 4, Supplementary Material |
| Selection process | 8 | Specify the methods used to decide whether a study met the inclusion criteria of the review, including how many reviewers screened each record and each report retrieved, whether they worked independently, and if applicable, details of automation tools used in the process. | Page 4 |
| Data collection process | 9 | Specify the methods used to collect data from reports, including how many reviewers collected data from each report, whether they worked independently, any processes for obtaining or confirming data from study investigators, and if applicable, details of automation tools used in the process. | Pages 4-5 |
| Data items | 10a | List and define all outcomes for which data were sought. Specify whether all results that were compatible with each outcome domain in each study were sought (e.g. for all measures, time points, analyses), and if not, the methods used to decide which results to collect. | Page 5 |
|  | 10b | List and define all other variables for which data were sought (e.g. participant and intervention characteristics, funding sources). Describe any assumptions made about any missing or unclear information. | Page 5 |
| Study risk of bias assessment | 11 | Specify the methods used to assess risk of bias in the included studies, including details of the tool(s) used, how many reviewers assessed each study and whether they worked independently, and if applicable, details of automation tools used in the process. | Page 5 |
| Effect measures | 12 | Specify for each outcome the effect measure(s) (e.g. risk ratio, mean difference) used in the synthesis or presentation of results. | Pages 5-6 |
| Synthesis methods | 13a | Describe the processes used to decide which studies were eligible for each synthesis (e.g. tabulating the study intervention characteristics and comparing against the planned groups for each synthesis (item #5)). | Pages 5-6 |
|  | 13b | Describe any methods required to prepare the data for presentation or synthesis, such as handling of missing summary statistics, or data conversions. | Pages 5-6 |
|  | 13c | Describe any methods used to tabulate or visually display results of individual studies and syntheses. | Pages 5-6 |
|  | 13d | Describe any methods used to synthesize results and provide a rationale for the choice(s). If meta-analysis was performed, describe the model(s), method(s) to identify the presence and extent of statistical heterogeneity, and software package(s) used. | Pages 5-6 |
|  | 13e | Describe any methods used to explore possible causes of heterogeneity among study results (e.g. subgroup analysis, meta-regression). | Pages 5-6 |
|  | 13f | Describe any sensitivity analyses conducted to assess robustness of the synthesized results. | Pages 5-6 |
| Reporting bias assessment | 14 | Describe any methods used to assess risk of bias due to missing results in a synthesis (arising from reporting biases). | Pages 5-6 |
| Certainty assessment | 15 | Describe any methods used to assess certainty (or confidence) in the body of evidence for an outcome. | Pages 5-6 |
| **RESULTS** | | |  |
| Study selection | 16a | Describe the results of the search and selection process, from the number of records identified in the search to the number of studies included in the review, ideally using a flow diagram. | Pages 6-7 |
|  | 16b | Cite studies that might appear to meet the inclusion criteria, but which were excluded, and explain why they were excluded. | Pages 6-7 |
| Study characteristics | 17 | Cite each included study and present its characteristics. | Pages 6-7 |
| Risk of bias in studies | 18 | Present assessments of risk of bias for each included study. | Pages 6-7 |
| Results of individual studies | 19 | For all outcomes, present, for each study: (a) summary statistics for each group (where appropriate) and (b) an effect estimate and its precision (e.g. confidence/credible interval), ideally using structured tables or plots. | Pages 6-7 |
| Results of syntheses | 20a | For each synthesis, briefly summarise the characteristics and risk of bias among contributing studies. | Pages 6-9 |
|  | 20b | Present results of all statistical syntheses conducted. If meta-analysis was done, present for each the summary estimate and its precision (e.g. confidence/credible interval) and measures of statistical heterogeneity. If comparing groups, describe the direction of the effect. | Pages 6-9 |
|  | 20c | Present results of all investigations of possible causes of heterogeneity among study results. | Pages 6-9 |
|  | 20d | Present results of all sensitivity analyses conducted to assess the robustness of the synthesized results. | Pages 6-9 |
| Reporting biases | 21 | Present assessments of risk of bias due to missing results (arising from reporting biases) for each synthesis assessed. | Pages 6-9 |
| Certainty of evidence | 22 | Present assessments of certainty (or confidence) in the body of evidence for each outcome assessed. | Pages 6-9 |
| **DISCUSSION** | | |  |
| Discussion | 23a | Provide a general interpretation of the results in the context of other evidence. | Page 9 |
|  | 23b | Discuss any limitations of the evidence included in the review. | Pages 11-12 |
|  | 23c | Discuss any limitations of the review processes used. | Pages 11-12 |
|  | 23d | Discuss implications of the results for practice, policy, and future research. | Pages 11-12 |
| **OTHER INFORMATION** | | |  |
| Registration and protocol | 24a | Provide registration information for the review, including register name and registration number, or state that the review was not registered. | Page 4 |
|  | 24b | Indicate where the review protocol can be accessed, or state that a protocol was not prepared. | Page 4 |
|  | 24c | Describe and explain any amendments to information provided at registration or in the protocol. | Page 4 |
| Support | 25 | Describe sources of financial or non-financial support for the review, and the role of the funders or sponsors in the review. | Page 1 |
| Competing interests | 26 | Declare any competing interests of review authors. | Page 1 |
| Availability of data, code and other materials | 27 | Report which of the following are publicly available and where they can be found: template data collection forms; data extracted from included studies; data used for all analyses; analytic code; any other materials used in the review. | Pages 1-2 |

*From:*  Page MJ, McKenzie JE, Bossuyt PM, Boutron I, Hoffmann TC, Mulrow CD, et al. The PRISMA 2020 statement: an updated guideline for reporting systematic reviews. BMJ 2021;372:n71. doi: 10.1136/bmj.n71

For more information, visit: <http://www.prisma-statement.org/>

# **SUPPLEMENTARY TABLE S2 - Search string for database search.**

PUBMED

| #5 | #1 AND #2 AND #3 AND #4 |
| --- | --- |
| #4 | "Diagnosis" OR "Detection" OR "Screening" OR "Sensitivity” OR “Specificity" OR "Accuracy"  OR “Diagnostic” |
| #3 | “Capsule Endoscopy"[Mesh] OR "PillCam" OR "Capsule” |
| #2 | "Colon" OR "Colorectal" OR "Colonic" OR “Intestinal” OR “Bowel” OR “Rectal” |
| #1 | "Polyp” OR “Lesion” |

EMBASE

| #5 | #1 AND #2 AND #3 AND #4 |
| --- | --- |
| #4 | 'diagnosis' OR 'detection' OR 'screening' OR 'sensitivity' OR 'specificity' OR 'accuracy' OR  'diagnostic' |
| #3 | 'capsule endoscopy':ti,ab OR 'pillcam':ti,ab OR 'capsule':ti,ab |
| #2 | 'colon':ti,ab OR 'colonic':ti,ab OR 'colorectal':ti,ab OR 'intestinal':ti,ab OR 'bowel':ti,ab OR  'rectal':ti,ab |
| #1 | 'polyp':ti,ab OR 'lesion':ti,ab |

CENTRAL

| #5 | "Polyp” OR “Lesion” |
| --- | --- |
| #4 | "Colon" OR "Colorectal" OR "Colonic" OR “Intestinal” OR “Bowel” OR “Rectal” |
| #3 | “Capsule Endoscopy"[Mesh] OR "PillCam" OR "Capsule” |
| #2 | "Diagnosis" OR "Detection" OR "Screening" OR "Sensitivity” OR “Specificity" OR "Accuracy"  OR “Diagnostic” |
| #1 | #5 AND #4 AND #3 AND #2 |

#

# **SUPPLEMENTARY FIGURE S1 - QUADAS-2 bias assessment for included studies in traffic light (A) and summary (B) graphs.**

# **
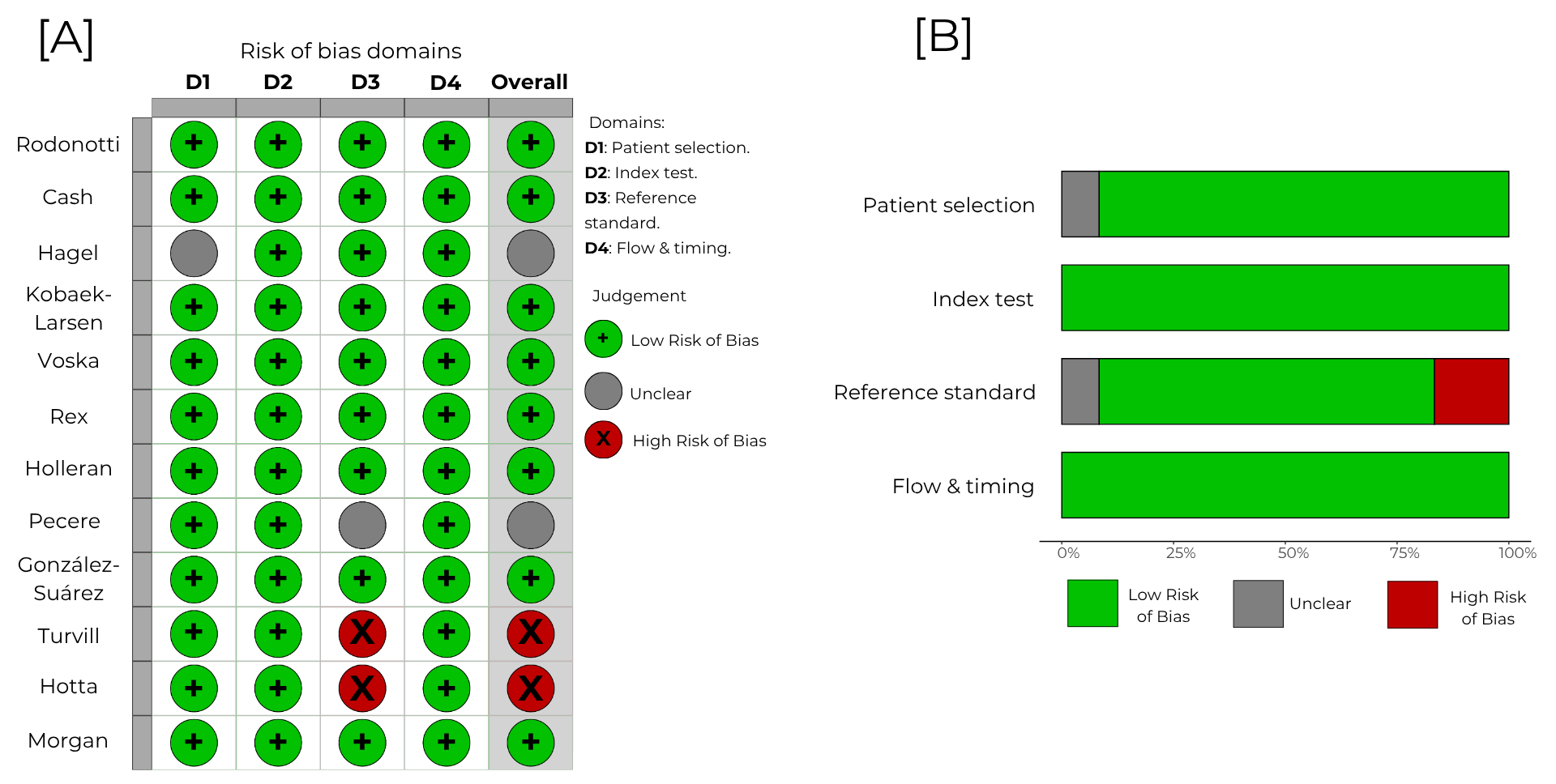
**

Both studies classified as high risk of bias received this judgment due to concerns in the reference standard domain. Specifically, in both cases, not all patients who underwent CCE-2 proceeded to follow-up colonoscopy, potentially introducing bias by selectively including patients with more advanced or complex lesions in the analysis.

# **SUPPLEMENTARY TABLE S3 - Moderator analyses for per-patient diagnostic metrics based on continuous moderators**

| Moderator | | p-value^a^ | | |
| --- | --- | --- | --- | --- |
|  |  | Any size | 6 mm or larger | 10 mm or larger |
| Adequate Preparation % | Sensitivity | 0.855^b^ | 0.436^c^ | 0.040^c^ |
|  | Specificity | NE | 0.223^c^ | 0.136^c^ |
| Complete Transit % | Sensitivity | NE | NE | 0.427^b^ |
|  | Specificity | NE | 0.127^c^ | 0.088^c^ |
| Male % | Sensitivity | NE | 0.537^b^ | 0.710^b^ |
|  | Specificity | NE | 0.774^b^ | 0.457^b^ |
| Mean Age | Sensitivity | NE | 0.427^c^ | 0.819^b^ |
|  | Specificity | NE | 0.234^b^ | 0.351^b^ |

^a^ Assessed through linear regression for linear models or analysis of variances (ANOVA) testing for restricted cubic splines. ^b^ Estimates yielded by linear models due to insufficient data for spline modeling (which we defined as either less than six points or less than three unique values). ^c^ Estimates yielded by restricted cubic splines with three knots, defined by the percentiles. Data that was not able to undergo either linear or restricted cubic spline regression due to insufficient data (which we defined as less than four points) was represented as *NE* (non-estimable).

# **SUPPLEMENTARY FIGURE S2 - Plot displaying the linear relationship between the sensibility of CCE-2 for the detection of polyps with 10 mm or larger and the proportion of adequate preparation in the included studies.**


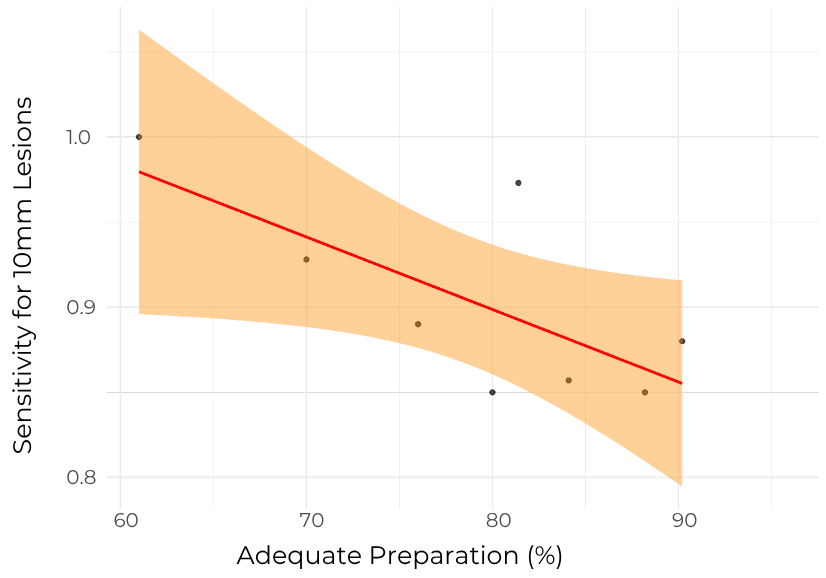


# **SUPPLEMENTARY FIGURE S3 - Funnel plots assessing publication bias based on diagnostic odds ratio for each size category.**


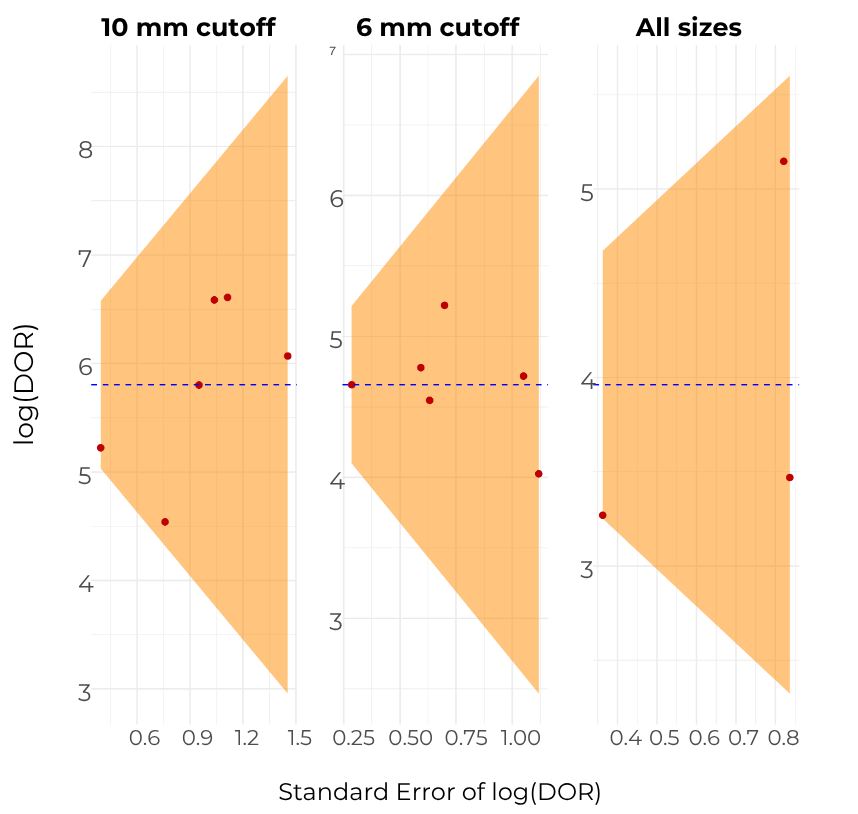


*DOR* diagnostic odds ratio.

# **SUPPLEMENTARY TABLE S4 - Data used for the construction of the simulated polyp screening cohort.**

|  | **Polyps of All Sizes** | **Polyps 6 mm or Larger** | **Polyps 10 mm or Larger** |
| --- | --- | --- | --- |
| **Predicted Sensibility (95% CrI)** | 0.90 (0.79 - 0.95) | 0.89 (0.85 - 0.92) | 0.91 (0.86 - 0.94) |
|  |  |  |  |
|  |  |  |  |
| **Predicted Specificity (95% CrI)** | 0.81 (0.66 - 0.91) | 0.94 (0.92 - 0.96) | 0.98 (0.96 - 0.99) |
| **Polyp Prevalence** | 62.7% | 33.5% | 18.2% |
| **PPV (95% CrI)** | 0.89 (0.82 - 0.95) | 0.88 (0.85 - 0.92) | 0.91 (0.84 - 0.96) |
| **NPV (95% CrI)** | 0.83 (0.71 - 0.93) | 0.94 (0.93 - 0.96) | 0.98 (0.97 - 0.99) |

We estimated predicted sensitivity and specificity from the posterior predictive distribution of our Bayesian model, simulating expected diagnostic performance in a new population. Polyp prevalence for each size category was calculated by aggregating patient-level data across studies that reported both the number of patients with polyps and the total number of patients, and then computing the corresponding proportions. Positive and negative predictive values (PPV and NPV) with 95% credible intervals were derived using a probabilistic approach. Sensitivity and specificity were modeled as independent beta distributions parameterized from the pooled means and 95% credible intervals, and 100,000 Monte Carlo samples were drawn. For each iteration, PPV and NPV were computed using standard Bayesian formulas incorporating disease prevalence. The 2.5th and 97.5th percentiles of the simulated distributions defined the 95% credible intervals
